# Implementation of Integrated Care in Singapore: A Complex Adaptive System Perspective

**DOI:** 10.5334/ijic.4174

**Published:** 2018-10-16

**Authors:** Milawaty Nurjono, Joanne Yoong, Philip Yap, Shou Liang Wee, Hubertus Johannes Maria Vrijhoef

**Affiliations:** 1 Center for Health Services and Policy Research, Saw Swee Hock School of Public Health, National University of Singapore and National University Health System, SG; 2 Center for Economic and Social Research, University of Southern California, California, US; 3 Khoo Teck Phuat Hospital, SG; 4 Geriatric Education and Research Institute, SG; 5 Panaxea B.V., Amsterdam, NL; 6 Department of Patient and Care, University Hospital Maastricht, Maastricht, NL; 7 Department of Family Medicine and Chronic Care, Vrije Universiteit Brussels, Brussels, BE

**Keywords:** integrated care, complex healthcare interventions, complex adaptive system

## Abstract

**Background:**

Integrated care that focuses on organising healthcare services around people and their communities rather than their diseases is promoted as the strategy to overcome the challenges associated with growing complexity in healthcare needs, demand for healthcare services and inadequate supply of services due to fragmentation in the provision of services. While conceptually appears to be simple, integrated care is made up of multicomponent delivery strategies targeting various levels of the healthcare system while engaging various stakeholders in their execution.

**Methods:**

We applied the complex adaptive system (CAS) perspective to two different initiatives that exemplify approaches towards integrating care in Singapore: the Regional Health System (RHS) model, implemented across healthcare institutions at the national level, and CARITAS Integrated Dementia Care implemented in the northern region of Singapore. We adopted an inductive approach in our analysis in which we studied the RHS and CARITAS Integrated Dementia Care according to the components of the CAS. We applied the typical characteristics of CAS: (i) diverse, interdependent and semi-autonomous actors (ii) self-organizing capacity and simple rules (iii) relationship with the bigger system, emergent behaviour and non-linearity in our analysis of key drivers behind the implementation of both the RHS and CARITAS integrated dementia care.

**Results:**

By considering the RHS and CARITAS as whole networks each comprising of interacting and adaptive components instead of separate entities within a bigger system, the CAS provided a new mind-set in surfacing issues associated to the implementation of these integrated care networks. In addition to important actors, systems, it informed understanding of relationships and dependencies between different parts of the network – revealing the lack of homogeneity, conformity and difficulties in designing any optimal system in advance given the many moving parts.

**Conclusions:**

Drawing on the two examples of integrated care networks, this paper highlights the significance of effective collaboration built on a common focus, responsiveness to emergent behaviours, simple rules, the ability to self-organize and adapt in response to unexpected situations in further development of integrated care in the Singapore context and beyond.

## Introduction

Today’s healthcare needs are becoming increasingly complicated, with rising demands for healthcare services and inadequate supply of services due to fragmentation in the provision of services. Addressing these pressures through care integration is a strategy that can help improve overall effectiveness, patient experience and sustainability [1]. The vision of integrated care is to place people and their communities at the centre of service provision, rather than their diseases. Although this conceptual premise appears simple, the World Health Organization (WHO)’s framework of people-centred integrated care emphasizes the complexity that typically underpins such interventions, highlighting the need to build multicomponent delivery strategies at all levels of the healthcare system while engaging various stakeholders in their execution [2], and considering the context of implementation [3].

Given the complexity of healthcare systems in general and models of integrated care in particular, except for a few [4,5,6,7], it is surprising to find that the journey towards integrated healthcare systems has hardly been examined through the lens of complex adaptive systems (CAS). In this descriptive paper, we applied the CAS perspective to two different initiatives that exemplify approaches towards integrating care in Singapore: the Regional Health System (RHS) model, implemented across healthcare institutions at the national level, and CARITAS Integrated Dementia Care implemented in the northern region of Singapore. We adopted an inductive approach in our analysis in which we studied the RHS and CARITAS Integrated Dementia Care according to the components of the CAS. We applied the typical characteristics of CAS [8], namely: (i) diverse, interdependent and semi-autonomous actors (ii) self-organizing capacity and simple rules (iii) relationship with the bigger system, emergent behaviour and non-linearity in our analysis of key drivers behind the implementation of both the RHS and CARITAS integrated dementia care. We discuss these characteristics and their implications below.

### Epidemiological Transition in Singapore

Like other developed nations, Singapore’s population is experiencing rapid aging, with a concomitant change in the prevalence and nature of chronic diseases. As of 2016, the median age of the Singapore’s population has increased to 40 years old in which 12.4% was found to be over 65 years old [9] and 16% were identified to have more than one chronic condition. The number citizen population aged 65 years or older is growing and estimated to triple to 900,000 [10]. Increasing prevalence of multi-morbidity among the aging population exerts significant burdens on the individuals, family, society as well as the healthcare system. Multi-morbidity reduces individuals’ capacity to seek help and self-manage, while simultaneously rendering service delivery more complex [11], leading to high consumption of healthcare resources [9] as outcomes grow worse – increased rates of further morbidity and resulting mortality [12], functional decline [8] and poorer quality of life [13].

### Singapore Healthcare System

Compromised of a network of public primary care clinics, public hospitals, tertiary-specialist care centres, private hospitals and non-government entities, Singapore healthcare system was designed with an emphasis on providing episodic care within acute hospitals in a largely disease centric manner and controlling infectious disease in a young population [14,15]. The Ministry of Health (MoH) takes full responsibility of the healthcare system. It planned, built and continues to develop and maintain the nation’s public healthcare system through governance, national healthcare services planning, structuring of healthcare financing and regulations [16].

Today, healthcare services are provided in Singapore through a mixed delivery model. The public sector dominates the hospitals, delivering 80% of the national burden of acute care. Meanwhile, primary care is largely provided by private general practitioners clinics whereas intermediate and long term care (ILTC) are primarily delivered by non-profit organizations, most of which are funded by the Government for their services rendered to patients [16,17]. Unlike other developed countries in which long term management of individuals with chronic illness is typically taken up by primary care providers within the community, chronic long-term care is largely delivered by acute and tertiary hospitals [15].

Healthcare financing in Singapore is built on the twin philosophies of individual responsibility and affordable basic healthcare for all. Healthcare is funded through a combination of government subsidies, multilayer financing schemes and co-payments through private individual savings, co-private insurance, employer medical benefits and out of pocket (OOP) payments [17]. Services are typically charged to patients based on disease, service and provider type with direct reimbursement from health savings accounts, supplier subsidies or out of pocket payment.

### Integrated Care Initiatives in Singapore

As the demands for healthcare services increase with aging, it is apparent that a disease-centric provision of services within the hospitals are becoming irrelevant, insufficient and unsustainable in the longer term [15]. This has prompted the realization that fundamental changes are urgently needed in order to refocus on prevention, primary care and community-based management, by breaking down silos and bringing the principles of integrated health and social care to service delivery, human capital management, financing and other organisational, policy and power structures that are at the risk of becoming irrelevant [18]. Two different initiatives towards integrated care in Singapore are:

- Regional Health System (RHS): The RHS model was introduced by the Ministry of Health (MOH) in 2012 to foster integrated care within respective geographic regions. Every RHS consists of a network led by a major public hospital, working in close partnership with other healthcare providers (primary care providers, community hospitals, nursing homes, home care and day rehabilitation providers) and social care providers within the same geographical region [18]. Each is provided with the mandate and funding support to plan and implement programmes that leverage their own network to provide healthcare beyond the hospital to the community, enabling the delivery of holistic, value-driven care across the entire continuum of care [19]. A dedicated strategic planning office in each RHS works to support the common vision of developing new care and financing models together with national agencies and key stakeholders, to facilitate integration of services across care settings, and to enable delivery of patient-centered care and appropriate siting of patients [personal communication]
- CARITAS Integrated Dementia Care: To meet the growing and multiple needs of persons with dementia (PWD) and their caregivers that go beyond medical needs in the north of Singapore, the clinical team of the Khoo Teck Phuat Hospital (KTPH) founded CARITAS integrated dementia care as an initiative within the northern RHS to improve care integration with the ultimate goal of providing comprehensive, accessible, responsive, individualized, trans-disciplinary (crosses different disciplines), accountable and seamless care for PWD [20]. Putting PWD and their families at the center of its efforts, the CARITAS integrated dementia care focuses on (i) enhancing the capacity, efficiency and quality of dementia care through vertical and horizontal care integration with team based care through regular case conferencing, shared and transfer of care from KTPH to primary and community care, (ii) improving competency and capability of primary and community care providers to provide dementia care through trainings, shared care and case conferencing and (iii) empowering caregivers through trainings and help-line.

## Applying a complex-adaptive system (CAS) perspective to the evolution of integrated care

Given the typical depth and breadth of needs driving any one intervention, the intricacy of many intervention components and the involvement of numerous actors with different perspectives and agendas within the integrated care setting, the implementation experience of such interventions can rarely be comprehensively or even meaningfully captured by a linear narrative of cause-and-effect.

For this reason, a complex-adaptive system (CAS) perspective has been increasingly advocated for categorizing and analyzing information in a manner that provides a more complete picture of forces affecting change around dynamic systems such as integrated care [7,21–23]. The CAS presents as a new perspective to guide the design, development and evaluation of integrated care systems through understanding key focus areas to allocate resources so as to achieve best possible outcomes [4,5,7,22,23].

Instead of being regarded (implicitly or explicitly) as a rigid controlled/controllable machine that produces predictable outcomes, a CAS is characterized as an open emergent and self-organizing system, made up of diverse, interdependent and semi-autonomous actors whose interactions are unpredictable and unplanned, therefore requiring successive adaptations [8,24,25]. With loose control and ability to re-organize within the environment in which it is set, the interactions between various actors in turn, shapes the behaviour of actors and the system as a whole.

Since their initiation, both the RHS model and CARITAS integrated dementia care have made significant progresses in developing the infrastructures for integrated care in Singapore. However, as may be expected, the journey to date has not been simple or linear in nature, as implementation challenges surface or evolve and outcomes related to these integrated care strategies remain uncertain. Furthermore, sustainability and scaling up continue to be major concerns. We hypothesize that studying these networks using the CAS perspective offers a new, in-depth way to interpret patterns and principles and develop insights appropriate to understanding and responding to issues surrounding these interventions.

Below, we used a CAS framework to describe both the RHS model and CARITAS integrated dementia care by focusing on: (i) the nature and interactions of the actors (ii) the networks’ simple rules and ability to self-organize (iii) the relationship of the networks to the bigger health system and (iv) non-linear emergent behaviours of the network. Then, insights about current implementation related to these themes were derived.

### Diverse, interdependent and semi-autonomous actors

**Figure 1:**
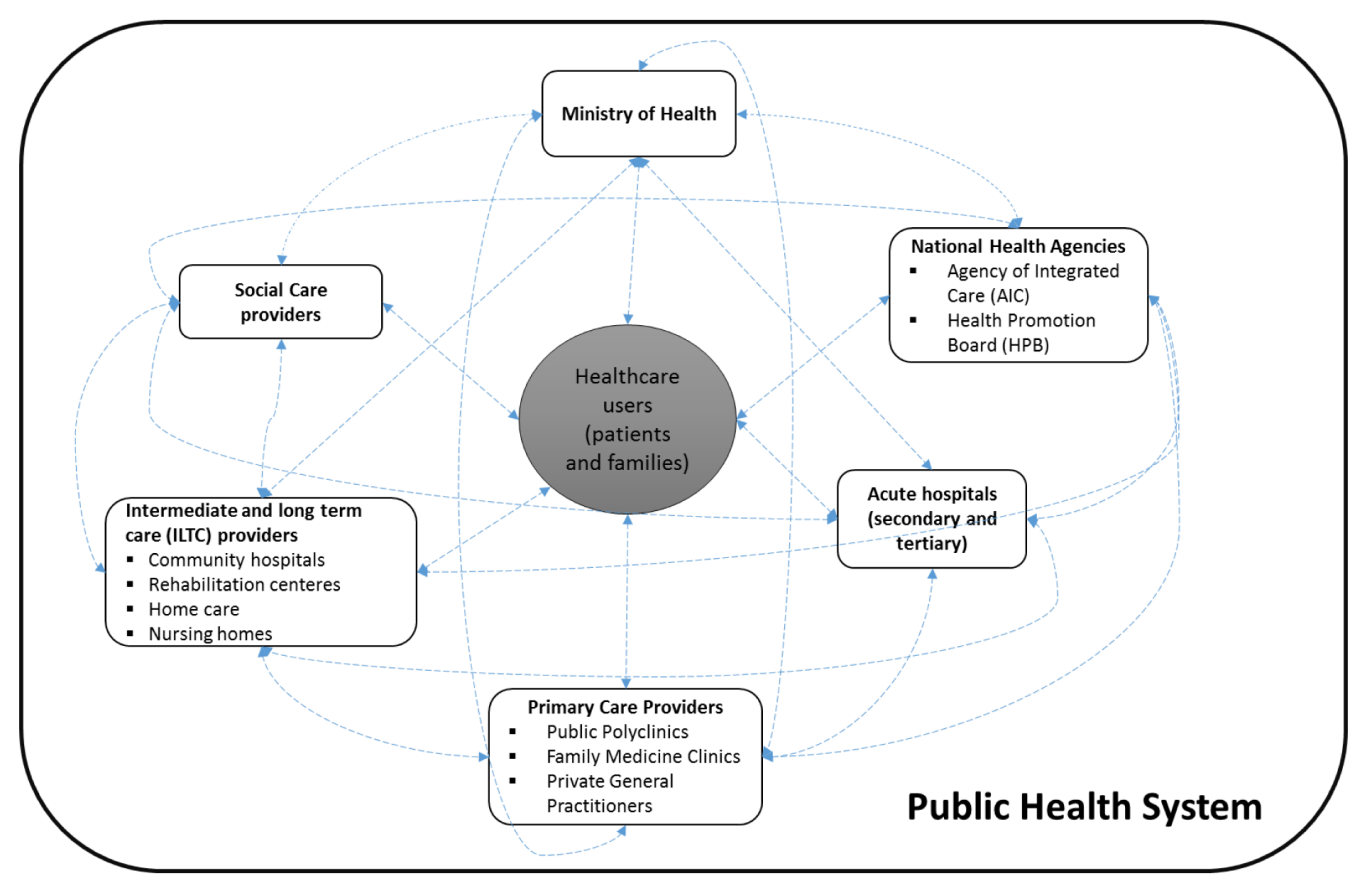
Representation of the RHS and CARITAS integrated dementia care as complex adaptive system (CAS).

Collectively, a wide range of actors within the RHS and CARITAS integrated dementia care as illustrated in Figure 1 take up different roles and contribute expertise towards the mandates and operations of the networks. Networks are defined by a clear delineation of geographical catchment areas as well as the common needs of the population in which the networks serve, leading to a diverse array of stakeholders.

Key actors involved in both the networks include: (i) Ministry of Health (MOH); (ii) two large healthcare-related government agencies, namely (a) the Agency for Integrated Care (AIC) which supports integration across the entire long-term care sector, as well as between primary, step-down and hospital care, and (b) the Health Promotion Board (HPB), which drives national health promotion and disease prevention programmes; (iii) primary care providers including public polyclinics and private general practitioners (GPs); (iv) secondary- and tertiary-care hospitals; (v) other intermediate and long-term care (ILTC) providers including community hospitals, day rehabilitation and care centres, home care/home nursing providers, and nursing homes; (vi) social and community partners; and (vii) healthcare users and their families. Although MOH is the primary funder and steward of the entire healthcare system, these actors interact along the care continuum with very few exclusive dyadic relationships and varying levels of autonomy.

In the case of integrated care, a collaborative mindset, coupled with strong internal and collective capabilities among organizations across all levels and roles are essential for success [26]. However, in a CAS, diversity can foster productive creativity but can also generate difficulties due to conflicting agendas, working styles, expectations, capacities and dynamics. Singapore is no exception. Leadership and funding is by design concentrated within the hospitals, an issue that is exacerbated by financial and human resource constraints at the community level. This imbalance is perpetuated by the longstanding perception among stakeholders that community-based care is inferior to hospital-based care, as well as large subsidies for public hospital-based care that distort the relative value of community care. In addition, the formal organization and practices within primary and community care in Singapore are less developed in comparison to the acute and secondary care sectors, as well as to the other counterparts in other developed countries [27], affecting their capability and capacity to collaborate. The capacity to collaborate at this level can also be relatively stretched, as private General Practitioners (GPs) and community care providers run as non-profits, with relatively thin operational budgets and margins. As a result, the introduction of new models of care can be challenging, as incentives may not always be aligned across patients and providers.

In such a setting, social capital and trust is critical and formed the basis of collaborations of both networks. However, the level of different partnerships can be highly volatile with time depending on whether collaborative relationships are bounded by explicit or implicit agreements. Partnerships are often influenced by changes in leadership, agendas, organization dynamics as well as available resources, and cannot be easily determined. On the one hand, there is little engagement of healthcare users in the design of the integrated care activities under the RHS, sometimes contributing to poor responses to the interventions. On the other hand, the engagement levels of patients and family members within CARITAS integrated dementia care also fluctuates with time and schedules especially with changes in PWD’s disease condition and social support. Other challenges manifest across provider relationships. As long-term careers in community care are perceived to be unattractive, this leads to a high individual turnover rate within community care organizations, in turn adversely affecting the continuity and stability of partnerships within the networks.

### Simple rules and self-organization capacity

Typically, a CAS is loosely controlled and constantly organizes itself in response to the environment in which it is set. Attempts to exert control over the whole are usually futile and restrict the system’s ability to react, adapt and innovate. Instead of rigid structures, simple rules/guiding principles may be more conducive to encouraging changes in a desired direction [8].

In both examples above, the MOH provides high-level funding and governance in the form of oversight and accountability for defined performance indicators. At the same time, their formation and operation embody very different approaches – top-down and bottom up, respectively to allocate appropriate resources, develop protocols and deliver care.

The RHS model was conceptualized by policymakers and implemented by healthcare providers. Key priority areas were determined by the MOH with very little contribution from healthcare users (patients and caregivers). Each of the 6 individual RHS formed across Singapore was tasked to implement the priority programs and held accountable for a jointly-agreed upon common set of outcomes. While the formation of the RHS reflects a desire for top-down control, in practice the successful operation of each RHS itself reflects considerable self-organisation, with each RHS planning office ensuring priority programmes are tailored to the unique needs and demographic of each population as well as the different strengths of community partners within its network, including overseeing the direct provision of services where capacity and capability remain nascent in the community. The planning office does not explicitly attempt to exert control over all collaborators, but instead simple rules including selection criteria, workflows and care protocols are jointly developed with the relevant actors of the networks to guide programme implementation. Beyond these guidelines, stakeholders are explicitly encouraged to innovate as long as any divergence falls within the working scope of population health for RHS. However, unintentionally the constraints of the national framework may limit the degree of risk-taking and experimentation.

By contrast, the very emergence of the CARITAS network reflects productive self-organisation and is driven by the clinical team within a tertiary hospital. The unmet needs of PWD and their families were first assessed and formed the basis for a new, team-based care mode that was conceptualized and proposed to MOH for funding. Multidisciplinary meetings involving different actors and cross disciplinary trainings continue to be held regularly to facilitate collaborations between different actors as well as to enhance capacity and capability of the network as a whole. Specialists are co-located within community healthcare settings and tele-consultations are conducted to provide decision support for primary and community care providers. Besides service providers, family members of PWD are actively engaged in the development of care plans and delivery of care for the PWD.

One challenge that exemplifies issues related to the imposition of rigid structure is the lack of adaptability of the healthcare financing system. Services are typically charged to patients based on disease, service and provider type with direct reimbursement from health savings accounts, supplier subsidies or out of pocket payments. There is currently no simple mechanism by which public sector providers themselves are able to pool charges across services and sectors. The development of simple, self-organized financing systems across the network or implementation of potentially transformative innovations in financing such as capitation models, portable subsidies, or bundled payments is therefore slow and highly limited to date.

### Embedded co-evolutionary system, non-linearity and emergent behaviour

Any CAS is embedded within and interacts with other systems. Both the RHS and CARITAS form part of the public healthcare system but are also embedded within a larger health and social care system that traverses different sectors and care settings. Interactions between different systems maybe be planned but often happen randomly in a non-linear fashion and gives rise to emergent behaviour which demands successive adaptations.

One example of planned interaction across systems is the introduction of a common information system, the National Electronic Medical Record. In principle, it forms an integrated virtual and long-term healthcare record centred on the patient, and is accessible to all authorized healthcare professionals across care settings and sectors who are a part of the network. While this is expected to improve information continuity, in practice, however, adoption remains a work in progress. The precision and completeness of documentation have been issues of concern. Moreover, difficulties faced in using the system, resistance to introduce new technology, and regulations around the use of personal information pose further barriers. In addition, the expectation to innovate quickly, yield positive outcomes within a short time and competition for public healthcare funding exert significant pressures on the healthcare providers and organizations. The multi-component interventions delivered by RHS and CARITAS require substantial time and resources for development, implementation and to show outcomes. Even when good results are demonstrated, given other competing programmes, continued funding is not guaranteed. Altogether, these contribute to frustrations among healthcare providers and in turn may adversely affect their participation in such complex interventions.

Besides the interactions between healthcare providers from different organizations, it is also important to consider the interaction between healthcare providers and users in the delivery of integrated care. While it is ideal for all actors to share a common view of how care should be organized and delivered, a mismatch in the expectation and perception of integrated care between healthcare providers and users has been reported in Singapore [28] where users showed little appreciation of the need for team-based care, patient-centeredness and the connectedness between social and healthcare needs. As such, implementation and uptake of integrated care programmes has been challenging due to low acceptability and adherence. In response, there are increasing efforts to engage healthcare users (patients and families) to improve acceptability of integrated care in Singapore. For instance, the CARITAS integrated dementia care network takes ownership of every person under its care. The network educates healthcare users on how care is delivered, the available support, ways to access services and self-management skills. Furthermore, when necessary, patients under the network are also given facilitated and expedited access to services.

Another non-linearity that must be recognized is related to the nature of illnesses as disease trajectories may unfold in an unpredictable way. Despite best efforts, the condition of the patient can take an unexpected turn for the worse. In this instance, providers need to adapt to the changing needs and work together with users to revise goals and work towards what is best for the patient and family. To this end, patient centredness would ideally entail shared decision making to empower the care recipients make more autonomous decisions under the guidance of healthcare providers. However, there remains a prevailing notion that healthcare providers “know best”, especially among older Singaporeans who are often passive in medical decision making (manuscript in preparation). Therefore, patient centeredness remains an aspirational goal which we can be optimistic will improve with succeeding cohorts of better informed and educated Singaporeans.

## Discussion

Integrated care is relatively new in Singapore and the progress with regards to a mind-set change towards collaborative care was reported to be slow-moving [29]. While applying CAS in full requires comprehensive data collection and analysis, here, we aimed to illustrate potential new insights with a CAS lens.

By considering the RHS and CARITAS as whole networks each comprising of interacting and adaptive components instead of separate entities within a bigger system[6], the CAS provided a new mind-set in surfacing issues associated to the implementation of these integrated care networks. In addition to important actors, systems, it informed understanding of relationships and dependencies between different parts of the network – revealing the lack of homogeneity, conformity and difficulties in designing any optimal system in advance given the many moving parts. This implies that, if incorporated early in design phase of an integrated care program, the CAS would help implementers view a new program as an adaptive system and not a machine, in which its functions and outcomes are not always predictable and require successive adaptations. With such a new perspective, implementers can then be guided to focus on the key determining factors of the CAS as mentioned above, increasing the possibility of more productive relationships and eventually better care outcomes.

We identified significant progress in the process of fostering integrated care within the RHS and CARITAS. Both networks provided platforms for different providers to collaborate and leverage on each other’s strengths. However, while different perspectives could foster greater productivity and creativity, the unique interplay of diverse actors within the network often complicate the implementation of the networks. Acknowledging that relationships and dependencies of actors change with different agendas and working styles that may also evolve with time, continual and active engagement of differential actors not to homogenize perspectives but to achieve a common focus is essential for the success of implementation of integrated care networks. Such engagement needs to be built on an understanding of common goals, roles, commitment, strengths and [26]. Besides these, engagement should also be operationalized to gather feedbacks to collectively improve the implementation efforts.

On the other aspect, commonly occurring non-linear interactions between providers and care recipients called for an increased responsiveness to emergent behaviours encountered. This can be achieved through active assessments of care recipients’ needs, preferences and subsequently involve them in shared decisions making to empower care recipients to make more autonomous decision. Understanding of patients’ needs and expectations of their health, abilities and constrains of the healthcare delivery is expected to enable providers to help care recipients achieve their best possible outcomes and experiences [30]. Nonetheless, in the context of Singapore, where providers and care recipients share a paternalistic relationship in the management of health, it may be necessary to proactively engage care recipients to provide them with essential knowledge about the network and their expected roles in the early phase of implementation.

Simple rules including selection criteria, workflows and care protocols jointly developed with the relevant actors of the networks provided useful guides for implementation of both networks. Meanwhile, the emphasis to self-organize has inspired and yielded innovations. Nevertheless, the unintentional constraints associated with rigidity and constant changes of the national framework had limited the degree self-organization. Often, this too created frustrations among healthcare providers, lowering their morale and responsiveness. It is therefore as important to build an environment that can support further development of the RHS and CARITAS as a CAS. In doing so, Sturmberg and Lanham proposed for a complex adaptive policy framework with loose boundaries that facilitate adaptability and allow emergence of optimal solutions best fitted for each unique care landscape [30]. Furthermore, at the policy level, primary and community care providers could be empowered to foster better partnerships, and by making changes to the current healthcare financing model to support team-based care and integrated care interventions. Instead of the traditional “fee for service” model, “fee for performance/complexity” can be explored to pay providers and organizations based on the complexity of illness and on patient outcomes. Rather than charging patients based on episodes of care and provider type, a financing mechanism that follows the patient and incentivizes quality and continuity of care should be put in place to pool charges across services and providers.

Finally, as changes surrounding the networks are inevitable, it is important for integrated care networks to increase in their adaptive capacity while constantly keeping the goal of an integrated care ecosystem a priority.

## Conclusion

Drawing on the two examples of integrated care networks, this paper highlights the significance of effective collaboration built on a common focus, responsiveness to emergent behaviours, simple rules, the ability to self-organize and adapt in response to unexpected situations in further development of integrated care in the Singapore context and beyond.
